# Outcomes of tranexamic acid administration in military trauma patients with intracranial hemorrhage: a cohort study

**DOI:** 10.1186/s12873-020-00335-w

**Published:** 2020-05-14

**Authors:** Patrick F. Walker, Joseph D. Bozzay, Luke R. Johnston, Eric A. Elster, Carlos J. Rodriguez, Matthew J. Bradley

**Affiliations:** 1 Department of Surgery, Uniformed Services University of the Health Sciences and Walter Reed National Military Medical Center, 8901 Rockville Pike, Bethesda, MD 20889 USA; 2 Department of Surgery, John Peter Smith Hospital, Ft. Worth, TX USA

**Keywords:** Tranexamic acid, TXA, Traumatic brain injury, TBI, Military

## Abstract

**Background:**

Tranexamic acid (TXA) may be a useful adjunct for military patients with severe traumatic brain injury (TBI). These patients are often treated in austere settings without immediate access to neurosurgical intervention. The purpose of this study was to evaluate any association between TXA use and progression of intracranial hemorrhage (ICH), neurologic outcomes, and venous thromboembolism (VTE) in TBI.

**Methods:**

This was a retrospective cohort study of military casualties from October 2010 to December 2015 who were transferred to a military treatment facility (MTF) in the United States. Data collected included: demographics, types of injuries, initial and interval head computerized tomography (CT) scans, Glasgow Coma Scores (GCS), and six-month Glasgow Outcome Scores (GOS). Results were stratified based on TXA administration, progression of ICH, and VTE.

**Results:**

Of the 687 active duty service members reviewed, 71 patients had ICH (10.3%). Most casualties were injured in a blast (80.3%), with 36 patients (50.7%) sustaining a penetrating TBI. Mean ISS was 28.2 ± 12.3. Nine patients (12.7%) received a massive transfusion within 24 h of injury, and TXA was administered to 14 (19.7%) casualties. Patients that received TXA had lower initial reported GCS (9.2 ± 4.4 vs. 12.5 ± 3.4, *p* = 0.003), similar discharge GCS (13.3 ± 4.0 vs. 13.8 ± 3.2, *p* = 0.58), and a larger improvement between initial and discharge GCS (3.7 ± 3.9 vs. 1.3 ± 3.1, *p* = 0.02). However, there was no difference in mortality (7.1% vs. 7.0%, *p* = 1.00), progression of ICH (45.5% vs. 14.7%, *p* = 0.09), frequency of cranial decompression (50.0% vs. 42.1%, *p* = 0.76), or mean GOS (3.5 ± 0.9 vs. 3.8 ± 1.0, *p* = 0.13). Patients administered TXA had a higher rate of VTE (35.7% vs. 7.0%, *p* = 0.01). On multivariate analysis, however, TXA was not independently associated with VTE.

**Conclusions:**

Patients that received TXA were associated with an improvement in GCS but not in progression of ICH or GOS. TXA was not independently associated with VTE, although this may be related to a paucity of patients receiving TXA. Decisions about TXA administration in military casualties with ICH should be considered in the context of the availability of neurosurgical intervention as well as severity of extracranial injuries and need for massive transfusion.

## Background

**Fig. 1:**
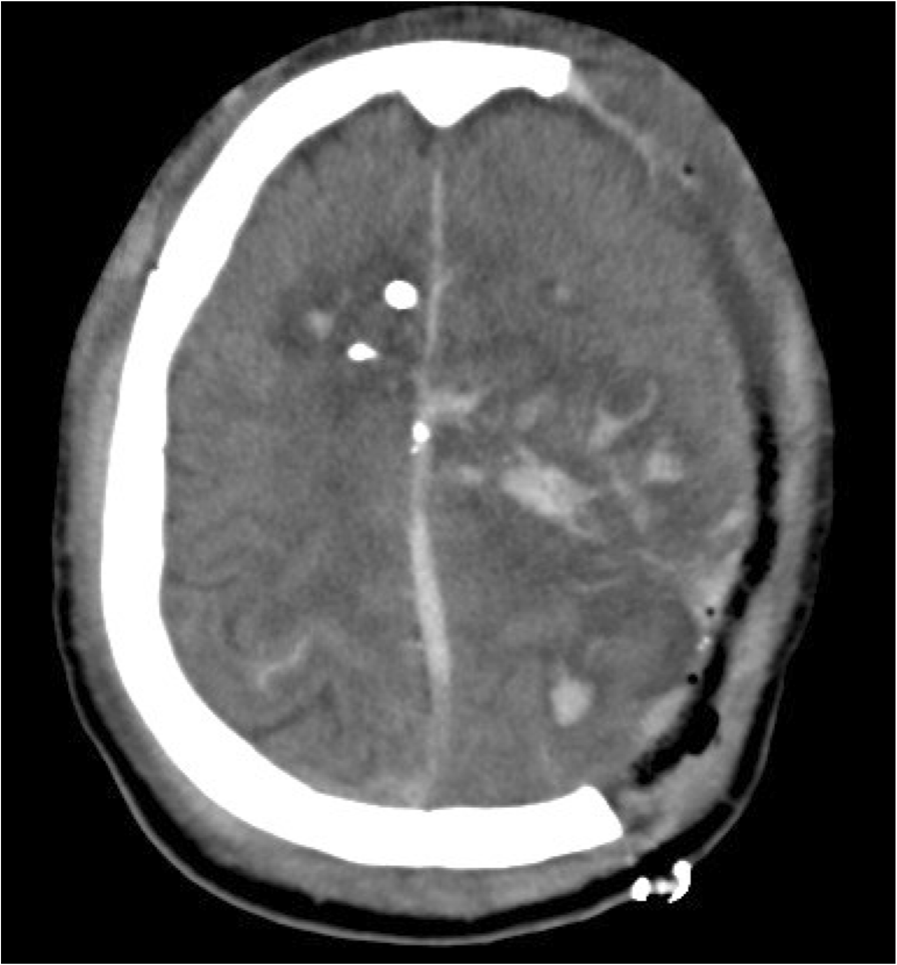
Computed tomography (CT) scan of patient with transhemispheric gunshot wound and associated intracranial hemorrhage

Tranexamic acid (TXA) may be a useful medication for severe traumatic brain injury (TBI) in austere settings without immediate access to neurosurgical intervention. This is commonly the case in combat, where TBI is often the result of penetrating injury and frequently requires neurosurgical intervention (Fig. 1) [1, 2]. Patients with TBI that require treatment in military settings, however, are often located in remote environments without immediate access to neurosurgical capability. In these circumstances, medical interventions are relied upon to mitigate causes of secondary brain injury, including hypotension, hypoxia, and hyperthermia. Although mass effect from exacerbation of intracranial hemorrhage (ICH) can also contribute to secondary brain injury, preventing this without surgical decompression can be difficult to accomplish [3].

Tranexamic acid (TXA), a medication that inhibits fibrinolysis by blocking the conversion of plasminogen to plasmin, has been theorized to decrease the progression of ICH [4]. The CRASH-2 study showed that If given within 3 h from the time of injury, TXA was associated with a survival advantage in civilian trauma patients with or at risk for significant bleeding [5, 6]. A survival advantage has also been demonstrated with TXA administration in military trauma patients [7]. However, the administration of TXA may be associated with thrombotic complications to include an increase in the incidence of venous thromboembolism (VTE) [8]. Current Department of Defense (DOD) Joint Trauma System (JTS) Tactical Combat Casualty Care (TCCC) Guidelines recommend TXA administration if a casualty is expected to need a significant blood transfusion and is able to be started no less than 3 h after injury, including in cases of hemorrhagic shock, one or more major amputations, penetrating torso trauma, or if severe bleeding is evident [9].

The outcomes of military patients with ICH who received TXA are not well described. Therefore, the purpose of this study was to evaluate any association between TXA use and progression of ICH as well as other outcomes including VTE in military patients with ICH. We hypothesized that TXA would not result in any difference in neurologic outcomes but would be associated with an increased incidence of VTE.

## Methods

This is a retrospective evaluation of all military casualties arriving to our military treatment facility (MTF) from October 1, 2010 to December 31, 2015 who were diagnosed with military combat-related ICH. This study was approved by the Walter Reed National Military Medical Center local institutional review board (protocol# 352329–27). Data was obtained from the Department of Defense Trauma Registry (DoDTR) in addition to the electronic medical record. The DoDTR collects and maintains information regarding the demographics, diagnoses, and interventions of combatant and noncombatant casualties from the point of injury until definitive care in the Continental United States (CONUS) at our Role IV MTF. Patients were excluded if they were not active duty United States service members or if they had incomplete data.

We collected information regarding the types of injuries and injury severity score (ISS), initial and interval head computerized tomography (CT) scans and differences between them, field and discharge Glasgow Coma Scores (GCS), and six-month Glasgow Outcome Scores (GOS) (Table 1). As shown in Table 1, the GOS defines neurologic outcomes 6 months after injury [10]. Patients who underwent craniectomy prior to a follow-up head CT were excluded when determining progression of ICH. Progression of ICH was determined qualitatively based on radiologist interpretation. Results were stratified based on TXA administration, ICH progression, and VTE occurrence.

**Table 1:** Glasgow Outcome Scale (GOS)

GOS 5	Resumption of normal life, minor deficits possible
GOS 4	Moderate disability without ADL assistance
GOS 3	Severe disability with ADL assistance
GOS 2	Persistent vegetative state
GOS 1	Death

For univariate analysis, statistical differences between continuous variables were determined by student’s *t* test for parametrically distributed data and by the Wilcoxon rank sum test for non-parametrically distributed data. Differences in categorical variables were determined by a continuity-adjusted chi-squared test or a Fisher’s exact test as appropriate. To evaluate independent clinical risk factors associated with VTE in our cohort, variables found to be statistically significant in univariate analysis were used to create a binary logistic regression model. A *p*-value < 0.05 was considered statistically significant. Statistical analysis was performed using SAS version 9.4 (SAS Institute Inc., Cary, NC).

## Results

687 active duty service members were treated at our facility during the study period. Of these, 71 patients had ICH (10.3%). Demographic data is shown in Table 2. 100% of patients were males. Most casualties were injured in a blast (80.3%), with 18.3% of casualties being injured from a gunshot wound. One patient had ICH from a closed head injury from a helicopter crash. 36 patients (50.7%) sustained a penetrating TBI, with 69.4% of those blast-related and the remaining 30.6% from gunshot wounds. Most patients had a mixed ICH pattern that included: 80.3% (*n* = 57) with intraparenchymal hemorrhage, 60.6% (*n* = 43) with subarachnoid hemorrhage, 46.5% (*n* = 33) with subdural hemorrhage, 25.3% (*n* = 18) with intraventricular hemorrhage, and 11.3% (*n* = 8) with epidural hemorrhage. Seven patients (9.9%) were reported to have diffuse axonal injury.

**Table 2:** Clinical characteristics of patients stratified by tranexamic acid administration. Continuous variables expressed as mean ± standard deviation

	Total (***n*** = 71)	TXA (***n*** = 14)	No TXA (***n*** = 57)	***P***-value
**Age, y**	25.2 ± 4.9	24.2 ± 3.1	25.5 ± 5.3	0.64
**ISS**	28.2 ± 12.3	36.6 ± 12.5	26.2 ± 11.4	0.003*
**Penetrating TBI, n**	36 (50.7%)	6 (42.3%)	30 (52.6%)	0.56
**Blast injury, n**	57 (80.3%)	12 (85.7%)	45 (80.0%)	0.72
**Gunshot wound, n**	13 (18.3%)	2 (14.3%)	11 (19.3%)	0.72
**Received massive transfusion within 24 h, n**	9 (12.7%)	4 (28.6%)	5 (8.8%)	0.07
**Platelets < 100 (×10**^**9**^**/L), n**	7 (9.9%)	3 (21.4%)	4 (7.0%)	0.13
**INR > 1.5, n**	14 (19.7%)	7 (50.0%)	7 (12.3%)	0.004*
**Lower extremity amputation, n**	6 (8.5%)	5 (35.7%)	1 (1.8%)	< 0.001*
**Pelvic fracture, n**	3 (4.2%)	1 (7.1%)	2 (3.5%)	0.5
**Chest AIS 3+, n**	17 (23.9%)	6 (42.3%)	11 (19.3%)	0.2
**Abdomen AIS 3+, n**	8 (11.3%)	3 (21.4%)	5 (8.8%)	0.19
**Extremity AIS 3+, n**	19 (26.8%)	8 (57.1%)	11 (19.3%)	0.008*
**Type of ICH**				
**Epidural, n**	8 (11.3%)	3 (21.4%)	5 (8.8%)	0.19
**Subdural, n**	33 (46.5%)	6 (42.3%)	27 (47.4%)	1.00
**Intraparenchymal, n**	57 (80.3%)	14 (100%)	43 (75.4%)	0.06
**Subarachnoid, n**	43 (60.6%)	8 (57.1%)	35 (61.4%)	0.77
**Intraventricular, n**	18 (25.3%)	6 (42.9%)	12 (21.1%)	0.17
**Presence of DAI, n**	7 (9.9%)	2 (14.3%)	5 (8.8%)	0.62

Mean ISS was 28.2 ± 12.3. Nine patients (12.7%) received a massive transfusion (> 10 units pRBC) within 24 h of injury, and TXA was administered to 14 (19.7%) casualties. Patients that received TXA were more severely injured (36.6 ± 12.5 vs. 26.2 ± 11.4, *p* = 0.003) (Table 2). They were also more likely to have had an INR > 1.5 (50% vs. 12.3%, *p* = 0.004) and lower extremity amputation (35.7% vs. 1.8%, *p* < 0.001). Four patients that were administered TXA (28.6%) did not have a chest, abdomen, or extremity abbreviated injury scale score of 3 or higher.

Patients that received TXA had lower initial reported GCS (9.2 ± 4.4 vs. 12.5 ± 3.4, *p* = 0.008), similar discharge GCS (13.3 ± 4.0 vs. 13.8 ± 3.2, *p* = 0.58), and a larger improvement between initial and discharge GCS (3.7 ± 3.9 vs. 1.3 ± 3.1, *p* = 0.02) (Table 3). However, there was no difference in mortality (7.1% vs. 7.0%, *p* = 1.0), mean 6-month GOS (3.5 ± 0.9 vs. 3.8 ± 1.0, *p* = 0.13), or need for cranial decompression (50.0% vs. 42.1%, *p* = 0.76).

**Table 3:** Outcomes stratified by tranexamic acid administration

	Total (***n*** = 71)	TXA (***n*** = 14)	No TXA (***n*** = 57)	***P***-value
**Underwent cranial decompression, n**	31 (43.7%)	7 (50.0%)	24 (42.1%)	0.76
**Field GCS**	11.8 ± 3.8	9.2 ± 4.4	12.5 ± 3.4	0.008*
**Discharge GCS**	13.7 ± 3.4	13.3 ± 4.0	13.8 ± 3.2	0.58
**Change in GCS**	1.8 ± 3.4	3.7 ± 3.9	1.3 ± 3.1	0.02*
**6-month GOS**	3.7 ± 1.0	3.5 ± 0.9	3.8 ± 1.0	0.13
**Mortality, n**	5 (7.0%)	1 (7.1%)	4 (7.0%)	1.0

26 patients (36.7%) underwent cranial decompression prior to their follow-up CT scan and therefore were excluded from determination of ICH progression to prevent confounding from postoperative changes. Of the 45 patients with an interval CT scan prior to intervention available for analysis, 11 (24.4%) demonstrated progression of ICH (Table 4). No differences in progression of ICH were noted between different types of ICH. There was no difference noted in presence of ICH progression in patients with penetrating TBI, thrombocytopenia, INR > 1.5, or in those who were administered TXA, although there was a trend towards a higher rate of progression in those patients that received TXA (45.5% vs. 14.7%, *p* = 0.09).

**Table 4:** Clinical characteristics stratified by progression of intracranial hemorrhage. ICH, intracranial hemorrhage; TBI, traumatic brain injury; INR, international normalized ratio; TXA, tranexamic acid

	Total (***n*** = 45)	ICH Progression (***n*** = 11)	No ICH Progression (***n*** = 34)	***P***-value
**Penetrating TBI, n**	13 (28.9%)	4 (36.3%)	9 (26.5%)	0.70
**Platelets < 100 (×10**^**9**^**/L), n**	5 (11.1%)	3 (27.3%)	2 (5.9%)	0.09
**INR > 1.5, n**	9 (20.0%)	3 (27.3%)	6 (17.7%)	0.67
**TXA administration, n**	10 (22.2%)	5 (45.5%)	5 (14.7%)	0.09

Nine patients in the cohort (12.7%) were diagnosed with VTE (Table 5). Patients with VTE had a higher mean ISS (40.3 ± 10.7 vs. 26.4 ± 11.5, *p* = 0.001) as well as more mean ventilator days (12.1 ± 5.2 vs. 5.7 ± 6.3, *p* = 0.004) and a higher mean of blood products transfused (44.2 ± 49.1 units vs. 13.4 ± 21.8, *p* = 0.008). They were also more likely to have had a lower extremity (66.7% vs. 24.1%, *p* = 0.02) or pelvic fracture (22.2% vs. 1.6%, *p* = 0.04) and to have been administered TXA (55.6% vs. 14.5%, *p* = 0.01). On multivariate analysis, however, TXA was not independently associated with VTE.

**Table 5:** Venous thromboembolism risk factors

	Total (***n*** = 71)	VTE (***n*** = 9)	No VTE (***n*** = 62)	***P***-value
**ISS**	28.2 ± 12.3	40.3 ± 10.7	26.4 ± 11.5	0.001*
**Lower extremity amputation, n**	6 (8.5%)	2 (22.2%)	4 (6.5%)	0.16
**Lower extremity fracture, n**	21 (29.6%)	6 (66.7%)	15 (24.1%)	0.02*
**Pelvic fracture, n**	3 (4.2%)	2 (22.2%)	1 (1.6%)	0.04*
**Spinal cord injury, n**	4 (5.6%)	1 (11.1%)	3 (4.8%)	0.43
**Ventilator days**	6.5 ± 6.5	12.1 ± 5.2	5.7 ± 6.3	0.004*
**Blood products transfused (units)**	17.3 ± 28.2	44.2 ± 49.1	13.4 ± 21.8	0.008*
**Received massive transfusion within 24 h, n**	9 (12.7%)	3 (33.3%)	6 (9.7%)	0.08
**Administered recombinant Factor VII, n**	1 (1.4%)	0 (0%)	1 (1.6%)	1.0
**Administered TXA, n**	14 (19.7%)	5 (55.6%)	9 (14.5%)	0.01*

## Discussion

In this study, we found that while there was an increase in GCS improvement with TXA administration in patients with military combat-related intracranial hemorrhage, there was no difference in progression of intracranial hemorrhage, need for cranial decompression, Glasgow Outcome Score, or mortality. Progression of intracranial hemorrhage has been associated with worse neurologic outcomes [11]. As traumatic brain injury is associated with coagulopathy from release of tissue factor [12–14], TXA can potentially decrease progression of intracranial hemorrhage and improve neurologic outcomes by counteracting this coagulopathy. A nested portion of the CRASH-2 study randomized patients with traumatic brain injury to receive TXA or placebo [15]. Although no differences were found regarding progression of ICH between the two groups, TXA use was shown to be safe in TBI patients. A placebo-controlled study from Thailand looking at TXA use in patients with intracranial hemorrhage also did not show a statistically significant change in size of the hemorrhage [4]. Prothrombin complex concentrate has been shown to decrease the growth of intracranial hemorrhage in geriatric trauma patients [16].

The potential use of TXA in for military TBI is of particular interest given the fact that the military is currently moving towards smaller, more remote surgical teams in order to support lower profile military operations [17]. Limited data is currently published about the effects of TXA in military combat-related TBI. Morte and colleagues published a propensity matched analysis looking at NATO patients with any type of head injury who received TXA [18]. They found a decreased mortality rate and improved discharge GCS in patients who received TXA. In comparison, in this study we looked specifically at patients with intracranial hemorrhage on CT scan and had access to six-month neurologic outcomes. While we also found an increase in GCS improvement in patients who received TXA, we did not see a benefit regarding mortality or Glasgow Outcome Score at 6 months. Additional data calls into question the potential benefit of TXA in military patients. In contrast to the MATTERs study, which showed that TXA was associated with a mortality benefit in military trauma patients [6], Howard and colleagues found in a review of 3773 casualties that TXA was not associated with a mortality difference [19].

The potential thromboembolic effects of TXA should be also be considered. In this study, we found that there was a significantly higher rate of TXA administration in patients with VTE. While our multivariate analysis was not significant, this finding is supported by other recent studies. Johnston and colleagues showed that TXA was an independent risk factor for VTE in military trauma patients [8]**.** A recent study of civilian trauma patients showed that TXA was associated with a more than threefold increase in the odds of VTE [20]. In both these studies, the increased risk of VTE is independent of injury severity, which was not the case in the MATTERs study. One potential reason for this is that the rate of VTE in MATTERs was only 4%, much lower than the 15.6 to 22.6% incidence reported in other series from the recent military conflicts [8, 21].

This study was limited by its retrospective nature and the constraints by which data was collected in the Department of Defense Trauma Registry. Patients were only included if they survived evacuation to the Continental United States, potentially excluding patients who had worse overall outcomes. We also did not have viscoelastic data to get a true sense for the presence of coagulopathy.

## Conclusions

Our study demonstrates that TXA may be associated with short-term neurologic improvement in military patients with intracranial hemorrhage but not in long-term neurologic outcomes or mortality. Additionally, TXA may be associated with an increased risk of VTE in this group. Based on our findings, we recommend that decisions about giving TXA to military trauma patients with suspected TBI should take into consideration extracranial injury severity as well as the proximity to neurosurgical capability for patients suspected to need cranial decompression.

## Data Availability

Available as requested from the corresponding author (patfwalker@gmail.com).

## Abbreviations

TXA: Tranexamic acid
TBI: Traumatic brain injury
ICH: Intracranial hemorrhage
VTE: Venous thromboembolism
MTF: Military treatment facility
CT: Computerized tomography
GCS: Glasgow coma score
GOS: Glasgow outcome score
ISS: Injury severity score
DOD: Department of Defense
JTS: Joint Trauma System
TCCC: Tactical Combat Casualty Care
DoDTR: Department of Defense Trauma Registry
INR: International normalized ratio
DAI: Diffuse axonal injury
